# May VelScope Be Deemed an Opportunistic Oral Cancer Screening by General Dentists? A Pilot Study

**DOI:** 10.3390/jcm9061754

**Published:** 2020-06-05

**Authors:** Stefania Leuci, Noemi Coppola, Anna Turkina, Maria Eleonora Bizzoca, Gianfranco Favia, Gianrico Spagnuolo, Michele Davide Mignogna

**Affiliations:** 1 Department of Neurosciences, Reproductive and Odontostomatological Sciences, Oral Medicine Unit, Federico II University of Naples, 80131 Naples, Italy; ste.leuci@gmail.com (S.L.); noemi.coppola@unina.it (N.C.); mignogna@unina.it (M.D.M.); 2 Department of Therapeuthic Dentistry, I.M. Sechenov First Moscow State Medical University (Sechenov University), 119991 Moscow, Russia; anna@turkin.su; 3 Department of Clinical and Experimental Medicine, University of Foggia, 71122 Foggia, Italy; marielebizzoca@gmail.com; 4 Interdisciplinary Department of Medicine, University of Bari “Aldo Moro”, 70121 Bari, Italy; gianfranco.favia@uniba.it

**Keywords:** VelScope, oral cancer, OSCC, pre-cancer, awareness, screening

## Abstract

Early diagnosis of oral cancer through visual inspection followed by histopathological confirmation is a pivotal step for reducing rates of morbidity and mortality. There are several auxiliary devices used to improve oral examination. The purpose of this cross-sectional pilot study is to evaluate the sensitivity and specificity of the Visually Enhance Lesion Scope (VelScope) system when it is used by the general dentist after a yearly oral medicine training. Thirty-five patients with oral lesions were evaluated with clinical and VelScope examination by two general dentists, one of which trained with a specific course. A comparison of the histopathological results, clinical examination, and VelScope made by both dentists was performed through statistical analysis. Sensitivity, specificity, positive predictive value (PPV), and negative predictive value (NPV) for detecting oral potentially malignant disorders (OPMDs) are 53.3%, 65%, 53.3%, 76.5% for unskilled dentist, 73.3%, 65%, 61.1%, 76.5% for skilled clinician. When both examiners use VelScope the values are 53.3%, 70%, 57.1%, 66.7% for unskilled general dentist (u-GD), 86.7%, 90%, 86.7%, 90% for skilled general dentist (s-GD). Improvement of a skilled general dentist for detecting malignancies is higher than inexperienced examiner when using VelScope. VelScope alone is unable to improve the general dentist’s ability to detect malignancies, but it could be a useful adjunctive device for clinicians when a focused training program is performed.

## 1. Introduction

Cancer is one of the most common causes of morbidity and mortality, with over 10 million new cases and over six million deaths each year in the world, and it is also responsible for 20% of all deaths in high-income countries and 10% in low-income ones [1]. Oral squamous cell carcinoma (OSCC) is a pathology still scarcely known by the general population, who is often unaware that the oral cavity may represent the place of the onset for malignant modifications. The symptoms are not interpreted correctly and are often ignored in the early stages of the pathological process. Despite the improvements in medical and surgical techniques, the prognostic data have not changed significantly over the last 10 years and are usually linked to the size of neoplasia and lymph node involvement: almost 85% of five-year survival for stages I/II, 25% at 5 years for stages III/IV, whereas the total five-year survival rate is around 50% [2]. It is clear that early diagnosis, therefore secondary prevention, is the only means to improve the prognostic pathway through the clinical oral examination (COE), where chromatic alteration, more or less accompanied by alterations of consistency and thickness of the tissue need to be detected. The oral cavity is an easily accessible area for COE with a reported sensitivity equal to 93% and a specificity equal to only 31% [3]. Still today, COE, together with histological examination, represents the “gold standard” for the definitive diagnosis. In literature, there are several auxiliary devices to improve COE, which should simplify the diagnostic process, such as Identafi, Narrow Band Imaging, Oral Cdx, ViziLite, and Visually Enhance Lesion Scope (VelScope) [4]. VelScope works through the emission of light with a wavelength of between 400–460 nm, which arouses the fluorophores in the oral epithelium. Normal tissue should show a normal green aspect on the VelScope examination (Vel-E), while dysplastic tissue should show darker aspects in comparison with the surrounding areas due to the loss of fluorescence (LAF) [5,6]. In the presence of LAF, the Vel-E is defined positive; it is due to several alterations, particularly: (i) break-up of the collagen cross-links, (ii) increase in blood supply due to micro-vascularization and inflammation, and (iii) reduction in flavine–adenine–dinucleotide (FAD) and nicotinamide-adenine-dinucleotide (NADH) (fluorochromes). These alterations could be present both in tissues with dysplasia and in tissues with ongoing inflammatory phenomena, thereby reducing the reliability of the test [7].

Many studies have highlighted several deficiencies in the knowledge and attitudes of primary health care dentists regarding OSCC [8,9]; therefore, strategies to improve diagnostic skills of general dentists are needed. In a new scenario, a cross-sectional pilot project was planned to explore the knowledge and clinical skills of general dentists (GDs) on the use of Vel-E, in cooperation with an oral medicine team. A one-year period of post-graduate oral medicine training was scheduled in order to improve clinical competence in patients with suspected oral lesions and discriminate benign oral diseases from oral potentially malignant disorders (OPMDs)/OSCC. The purpose of the project is to evaluate whether the VelScope system improves the diagnostic ability to detect OPMDs when it is used by inexperienced examiners.

## 2. Material and Methods

From December 2018 to November 2019, we asked two GDs to perform COE and Vel-E on consecutively selected patients during the first visit, one of which after a 1-year training program in oral medicine at the Oral Medicine Unit, Federico II University of Naples, Italy. All patients signed an informed consent before undergoing the project. The study was conducted in accordance with the Declaration of Helsinki, and the protocol was approved by the Institutional Ethics Committee of the University of Bari (N° 1442). The reporting of data followed the guidelines of the STARD statement.

The patients were first subjected to a complete clinical interview, and COE encompasses a manual and visual examination of intra- and extra-oral sites. Based on the clinical characteristics found, both the examiners classified the lesions into benign or malignant and performed Vel-E on the basis of clinical information and in the absence of histological data. The device was used, maintaining a distance of approximatively 5 cm from the oral cavity to optimize the visualization of the natural tissue fluorescence and, if necessary, darken the operating theatre to reduce natural light and bring it to an acceptable level. Subsequently, incisional or excisional biopsy was carried out by an oral medicine specialist with a histological examination by a pathologist at the Interdisciplinary Department of Medicine, University of Bari Aldo Moro, Italy. The margins of the incision are guided by the result of the Vel-E. All specimens were placed in 4% buffered formalin solution for fixation, embedded in paraffin and were cut into 4 μm thick sections, and stained with hematoxylin/eosin. The oral pathologist who examined the biopsy specimens was blind to the Vel-E. The time interval between the Vel-E and the biopsy examination was an average of 15 days; during this period, the patients did not perform any therapy. The results of the histological examination have been compared with those of the COE and VelScope.

The clinical photographic documents were obtained by using a Canon^®^ Eos 750D (Tokyo, Japan) camera with Canon^®^ EF-S 60 mm f/2.8 Macro lens, while the VelScope photographic images were produced with a digital camera (Canon) with a specific adaptor.

**Figure 1:**
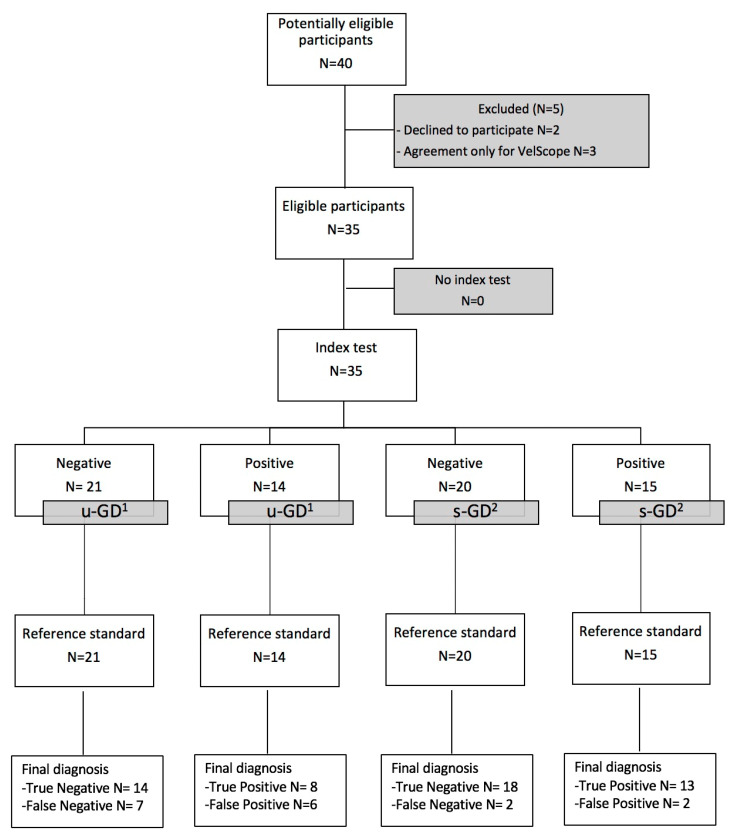
Flow chart of the study. ^1^ u-GD: unskilled general dentist; ^2^ s-GD: skilled general dentist.

Potentially eligible participants were identified on the basis of the following inclusion and exclusion criteria (Figure 1). The inclusion criteria were: 19–90-year-old patients of both genders, Caucasian, affected by OPMDs as leukoplakia and erythroplakia, oral lichen planus, lichenoid lesions, unspecified keratotic lesions (KUS). The exclusion criteria were patients affected by benign lesions and para-physiological anomalies (such us Fordyce granules, thickened Linea alba, white sponge nevus, hypertrophy of lingual tonsils and of the lymphoid tissue of Waldeyer’s ring, exogenous and endogenous pigmentations, prominent varices of the ventral lingual, morsicatio buccarum, benign alveolar ridge keratosis, geographic tongue, leukoedema).

A descriptive statistical analysis was produced through the assessment of sensitivity, specificity, positive predictive value (PPV), negative predictive value (NPV) of the Vel-E performed on oral mucosal lesions. Sensitivity and specificity, PPV and NPV of COE, and Vel-E were calculated using 2 × 2 contingency tables. Biopsy and histological examination were used as the gold standard.

## 3. Results

The clinical and demographic characteristics of the patients are shown in Table 1. Thirty-five patients, 17 males (48.6%) and 18 females (51.4%), with an average age of 58.5 years, were enrolled. The unskilled general dentist examiner (u-GD) classified as benign 20 oral lesions and as malignant 15 oral lesions, instead the skilled general dentist examiner (s-GD) 17 and 18, respectively.

**Figure 2:**
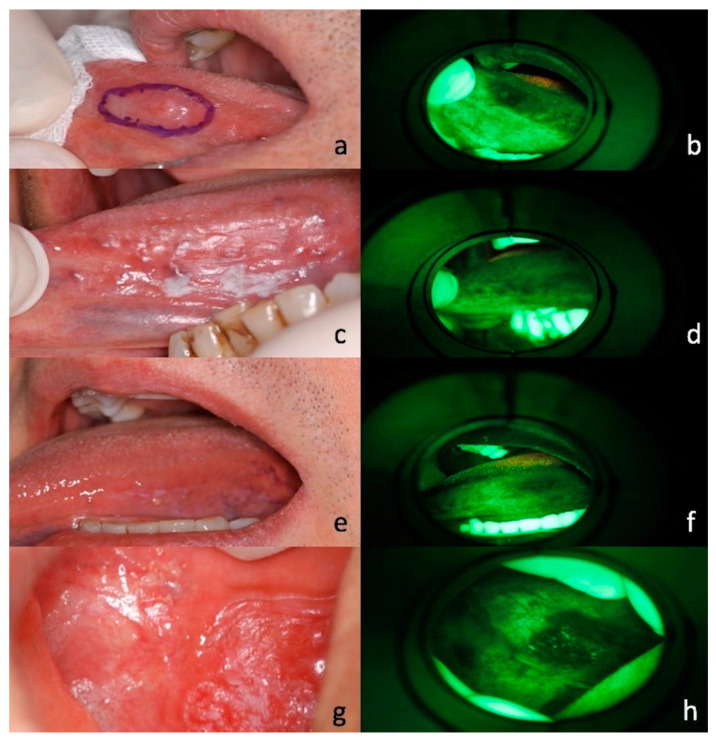
The image shows cases of loss of autofluorescence (LAF) on the VelScope examination on suspicious lesions that have been confirmed malignant on histological examination. (**a**,**b**) Clinical appearance and VelScope examination of nodular lesion of the left ventral tongue in a 58-year-old male smoker patient; (**c**,**d**) Clinical appearance and VelScope examination of single keratotic lesion of the left ventral tongue in a 47-year-old male patient; (**e**,**f**) clinical appearance and VelScope examination of keratotic and erythematous lesion of the left ventral tongue in a 52-year-old male patient; (**g**,**h**) clinical appearance and VelScope examination of erythematous speckled lesion of the buccal mucosa in a 65-year-old female patient.

**Figure 3:**
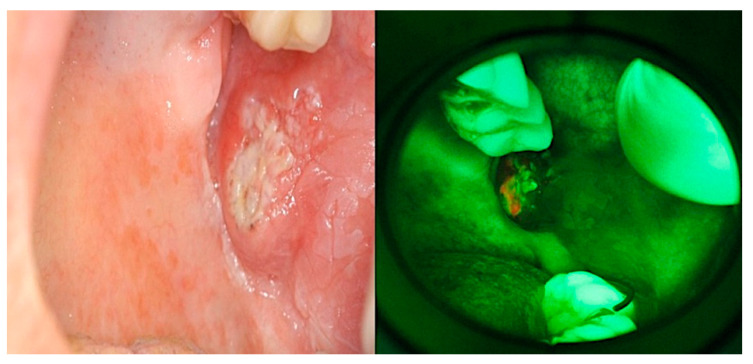
Clinical appearance of the painful suspected lesion on the right buccal mucosa in a 63-year-old female smoker patient and appearance of the VelScope exam of the same lesion characterized by LAF and associated red/orange coloring typical of secondary bacterial contamination.

The Vel-E was interpreted by u-GD as positive in 14 patients (40%) and negative in 21 patients (60%); instead, VelScope data from s-GD were positive in 15 patients (42.9%) and negative in 20 patients (57.1%) (Figure 2a–h). LAF showed a range of colors from dark green to black, while one case showed up orange/red due to bacterial contamination (Figure 3).

On histological examination, 15 (42.9%) of 35 biopsied lesions were malignant, and the other 20 (57.1%) were benign. The suspected clinical diagnosis (benign/malignant) made by both GDs and the VelScope data were compared with the histological results. As seen in Table 2, sensitivity, specificity, PPV and NPV of u-GD for detecting OPMDs are 53.3%, 65%, 53.3%, 76.5% respectively, while the same parameters for s-GD are 73.3%, 65%, 61.1% and 76.5% respectively. Differently, when both examiners use an adjunct device the values of sensitivity, specificity, PPV and NPV change in the following way: for u-GD they were 53.3%, 70%, 57.1%, 66.7%, conversely for s-GD they were 86.7%, 90%, 86.7% and 90% (Table 2).

We observed only two cases of false positives (5.71%) in s-GD and 6 (17.14%) in u-GD and two false-negative cases with minimal and poorly detectable LAF in s-GD, and 7 in u-GD.

## 4. Discussion

Benign and malignant oral lesions clinically appear with great variability in terms of heterogeneous morphology, onset, and clinical course; this is why histopathological examination remains the gold standard for final diagnosis. Up to now, there are unresolved medical issues in the methodology of published reports, due to selection bias of patients with different OPMDs and different prognosis and to the absence of uniformity in histological classification and description of the grade of dysplasia [10]. The heterogeneity of clinical and histological aspects among experts clearly reflects the diagnostic difficulty to screen oral lesions and does not allow data to be uniformly compared. Awan et al., in a previous study on 164 patients analyzed with Vel-E both lesions clinically suspected for malignancies and clinically benign showed a good sensitivity of 84% with a very low specificity equal to 15%, suggesting that data on specificity could be very different based on the inclusion criteria of the study group [11].

To deal with the issue of OSCC diagnostic delay, it is necessary to focus on early diagnosis and prevention systems in the general population [12]. Identification of high-risk individuals and screening systems are part of secondary prevention. An effective oncological screening system has the main goal to find cancer early and to reduce the possibility that patients screened regularly will die from cancer; it is also a preventive measure for a high-risk population. Not all types of cancer currently have an effective screening method, and there is no single approach that fits all situations [13]. There is currently no standard or routine screening test for OSCC; however, a visual inspection should be a routine step in oral health practitioner daily activity [14]. COE is characterized by a simple, non-invasive procedure, that is easy, effective, and cheap [15]. Nevertheless, the lack of evidence does not support COE as part of a population-based screening program, while it reduces the mortality rate of OSCC in “high-risk” patients [16]. The use of adjunctive tests or devices, such as toluidine blue, brush biopsy and fluorescence, can be useful in the evaluation of oral mucosal lesions [17], but it is not evidence-based as well as a useful tool to reduce OSCC mortality in the absence of specialist training [18]. Among the different screening systems described in the literature, VelScope is the one supported by the majority of published studies. The efficacy of VelScope in detecting OPMDs when used by professionals who are experts in the oral diagnosis and/or on lesions clinically suspected for malignancies is widely documented [19]. This is a key point of criticism because the use of VelScope now is “targeted”, usually applied on “high-risk” patients with OPMDs/OSCC and/or entrusted to trained clinicians [20]. Therefore, the high values of sensitivity and specificity reported have not been tested in the general dentistry field; for this reason, VelScope is not supported by robust evidence for its use in primary care, but only in selected specialist clinics where trained physicians may discriminate among OPMDs [21]. However, an effective screening system must be applicable in the context of primary care by all the physicians who take care of oral health (general/specialist dentists, otorhinolaryngologists, general physicians, maxillofacial surgeons).

Huff et al. reported in a retrospective pilot study the routine utilization of Vel-E in general dental practice on “low-risk” adults were showing that it could be useful in identifying OPMDs also in this different population setting [22]. We have tried to understand if a training focused course of continuing education on OSCC in general dental practice based on a one-year program with the specific clinical session can improve early diagnosis on high-risk patients when Vel-E is added to COE. Our analysis showed an increase of 13.4% and 25% for sensitivity and specificity, respectively, when s-GD used Vel-E in adjunction to COE. Instead, for u-GD, the sensitivity value was not changed, while the specificity value was increased by 5%. Thus, the improvement of s-GD’s efficacy for detecting OPMDs was more significant with the aid of fluorescence compared to u-GD’s. In our study, the use of autofluorescence increases sensitivity, specificity, PPV, and NPV for the s-GD in detecting suspected oral lesions compared to COE. These results are consistent with a similar project by Simonato et al., in which it proved a similar increase in the efficacy of Vel-E to detect early oral suspected lesions, but in this case, the main actors are represented by dental students versus expert professional in oral medicine [23].

One of the major problems using Vel-E is that it does not provide an objective result in evaluating color change of oral epithelium. Therefore, the threshold between loss of auto-fluorescence and mucosa with normal auto-fluorescence can be arbitrary, and it can often depend on the ability and experience of the operator. This issue limits the effectiveness of this device as cancer screening adjunct if used by inexperienced operators and reinforces the need for training courses [24]. Another critical aspect of the auto-fluorescence observed in literature is the presence of false positives. The percentage of false positives is much higher in groups of patients with reactive or not dysplastic alterations because many inflammatory lesions may show LAF due to the presence of vascular inflammatory modifications (hyperemia and vasodilatation) [25]. In our study, we observed only two cases of false positives (5.71%) in s-GD and 6 (17.14%) in u-GD. On the contrary, false negative are rarely reported in the literature; in our study, we had two false-negative cases with minimal and poorly detectable LAF in s-GD, and 7 in u-GD. The false negatives could be due to an unclear autofluorescence pattern considered negative by the examiners.

Therefore, the VelScope could be an adjunctive device for GDs in the screening of OSCC only when a focused training program is performed. From these data, we confirm the pivotal role of continuing oral medicine education to improve diagnostic skills of GDs for detecting oral “high-risk” lesions, and we highlight the need of free mandatory yearly post-graduate training courses in order to give more and more expertise and clinical skills to use screening system devices [26].

Indeed, the targeted-education is the reason for improving oral diagnosis by GDs and increases their awareness in referring patients to the specialists for early diagnosis, which remains the best tool to reduce OSCC mortality.

The study has, however, different limitations. First, the relatively small sample size both of the examiners and patients; second, the absence of objective autofluorescence visualization including an image analysis software to obtain semi-quantitative or quantitative values.

## 5. Conclusions

Data from this pilot study indicate that VelScope alone is unable to improve GDs’ ability to detect OPMDs, but it could be an aid for skilled oral practitioners so as to refer patients to oral medicine centers at an early stage of the disease. It would be necessary to educate non-experts on the correct use of adjunct devices whose interpretation of the results requires appropriate and specific training in oral medicine. Further studies with extended samples are necessary to assess the efficacy of VelScope screening on the general population and to determine if it might be useful to improve early diagnosis of OPMDs and OSCC by general dentists after adequate training.

## Figures and Tables

**Table 1:** Baseline demographic and clinical characteristics of participants.

Characteristics	Number (%)
**Age (mean year)**	58.5
**Gender**	
Male	17 (48.6%)
Female	18 (51.4%)
**Risk factor**	
*** Smoking status***	
Never	15 (42.9%)
Former smoker	7 (20%)
Current smoker	13 (37.1%)
*** Alcohol use***	
Never	21 (60%)
Former drinker	9 (25.7%)
Current drinker	5 (14.3%)
**Oral Lesions**	
** *COE ^1^ u-GD ^2^***	
Suspected malignant lesions	15 (42.9%)
Suspected benign lesions	20 (57.1%)
** *COE ^1^ s-GD ^3^***	
Suspected malignant lesions	18 (51.4%)
Suspected benign lesions	17 (48.6%)
** *VEL-E ^4^ u-GD ^2^***	
Positive	14 (40%)
Negative	21 (60%)
** *VEL-E ^4^ s-GD ^3^***	
Positive	15 (42.9%)
Negative	20 (57.1%)
**Histopathological diagnosis**	
** *Benign***	
OLP ^5^	9 (25.7%)
OLL ^6^	5 (14.3%)
Cheratotic lesion	2 (5.7%)
OL ^7^	4 (11.4%)
** *Malignant***	
Erythroplakia	4 (11.4%)
CIS ^8^	3 (8.6%)
OSCC ^9^	6 (17.1%)
Verrucous carcinoma	2 (5.7%)

^1^ COE: conventional oral examination; ^2^ u-GD: unskilled general dentist; ^3^ s-GD: skilled general dentist; ^4^ VEL-E: VelScope examination; ^5^ OLP: oral lichen planus; ^6^ OLL: oral lichenoid lesion; ^7^ OL: oral leukoplakia; ^8^ CIS: carcinoma in situ; ^9^ OSCC: oral squamous cell carcinoma.

**Table 2:** Sensibility, specificity, positive predictive value, negative predictive value for the conventional oral examination, and autofluorescence visualization for unskilled-general dentist and skilled-general dentist.

	u-GD ^1^ (COE ^2^)	u-GD ^1^ (Vel-E ^3^)	s-GD ^4^ (COE ^2^)	s-GD ^4^ (Vel-E ^3^)
**Sensitivity**	53.3% (26.59–78.73%)	53.3% (26.59–78.73%)	73.3% (44.90–92.21%)	86.7% (59.54–98.34%)
**specificity**	65% (40.78–84.61%)	70% (45.72–88.11%)	65% (40.78–84.61%)	90% (68.30–98.77%)
**ppv ^5^**	53.3% (34.78–71.01%)	57.1% (37.00–75.17%)	61.1% (44.55–75.45%)	86.7% (63.23–96.09%)
**npv ^6^**	76.5% (49.74–77.70%)	66.7% (52.02–78.68%)	76.5% (56.95–88.87%)	90% (71.07–97.06%)

^1^ u-GD: unskilled general dentist; ^2^ COE: conventional oral examination; ^3^ VEL-E: VelScope examination; ^4^ s-GD: skilled general dentist; ^5^ PPV: positive predictive value; ^6^ NPV: negative predictive value.
